# Thalidomide Reduces Cell Proliferation in Endometriosis Experimentally Induced in Rats

**DOI:** 10.1055/s-0039-3399551

**Published:** 2019-11

**Authors:** Luana Grupioni Lourenço Antônio, Julio Cesar Rosa-e-Silva, Deborah Juliani Machado, Andrezza Telles Westin, Sergio Britto Garcia, Francisco José Candido-dos-Reis, Omero Benedicto Poli-Neto, Antonio Alberto Nogueira

**Affiliations:** 1 Faculty of Medicine of Ribeirão Preto, Universidade de São Paulo, Ribeirão Preto, SP, Brazil

**Keywords:** experimental endometriosis, cell proliferation, PCNA, thalidomide, rat, endometriose experimental, proliferação celular, PCNA, talidomida, rata

## Abstract

**Objective** To analyze the effect of thalidomide on the progression of endometriotic lesions experimentally induced in rats and to characterize the pattern of cell proliferation by immunohistochemical Proliferating Cell Nuclear Antigen (PCNA) labeling of eutopic and ectopic endometrium.

**Methods** Fifteen female Wistar rats underwent laparotomy for endometriosis induction by resection of one uterine horn, isolation of the endometrium and fixation of a tissue segment to the pelvic peritoneum. Four weeks after, the animals were divided into 3 groups: control (I), 10mg/kg/day (II) and 1mg/kg/day (III) intraperitoneal thalidomide for 10 days. The lesion was excised together with the opposite uterine horn for endometrial gland and stroma analysis. Eutopic and ectopic endometrial tissue was submitted to immunohistochemistry for analysis of cell proliferation by PCNA labeling and the cell proliferation index (CPI) was calculated as the number of labeled cells per 1,000 cells.

**Results** Group I showed a mean CPI of 0.248 ± 0.0513 in the gland and of 0.178 ± 0.046 in the stroma. In contrast, Groups II and III showed a significantly lower CPI, that is, 0.088 ± 0.009 and 0.080 ± 0.021 for the gland (*p* < 0.001) and 0.0945 ± 0.0066 and 0.075 ± 0.018 for the stroma (*p* < 0.001), respectively. Also, the mean lesion area of Group I was 69.2 mm2, a significantly higher value compared with Group II (49.4 mm2, *p* = 0.023) and Group III (48.6 mm2, *p* = 0.006). No significant difference was observed between Groups II and III.

**Conclusion** Thalidomide proved to be effective in reducing the lesion area and CPI of the experimental endometriosis implants both at the dose of 1 mg/kg/day and at the dose of 10 mg/kg/day.

## Resumo

**Objetivo** Analisar o efeito da talidomida na progressão de lesões endometrióticas induzidas experimentalmente em ratas e caracterizar o padrão de proliferação celular pela marcação imunohistoquímica de Antígeno Nuclear de Célula Proliferativa (PCNA) no endométrio eutópico e ectópico.

**Métodos** Quinze ratas Wistar foram submetidas a laparotomia para indução de endometriose por ressecção de um corno uterino, isolamento do endométrio e fixação de um segmento do tecido ao peritônio pélvico. Após quatro semanas, os animais foram divididos em 3 grupos: controle (I), 10 mg/kg/dia (II) e 1 mg/kg/dia (III) de talidomida intraperitoneal por um período de 10 dias. As lesões foram resseccionadas juntamente com o corno uterino oposto para análise da glândula endometrial e do estroma. O tecido endometrial eutópico e ectópico foi submetido à imunohistoquímica para análise da proliferação celular por marcação com PCNA e o índice de proliferação celular (CPI) foi calculado como o número de células marcadas por 1.000 células.

**Resultados** O grupo I apresentou média de CPI de 0,248 ± 0,0513 na glândula e de 0,178 ± 0,046 no estroma. Em contraste, os grupos II e III apresentaram CPI significativamente menor, isto é, 0,088 ± 0,009 e 0,080 ± 0,021 para a glândula (*p* < 0,001) e 0,0945 ± 0,0066 e 0,075 ± 0,018 para o estroma (*p* < 0,001), respectivamente. Além disso, a área de lesão média do Grupo I foi de 69,2 mm^2^, valor significativamente maior em relação ao Grupo II (49,4 mm^2^, *p* = 0,023) e Grupo III (48,6 mm^2^, *p* = 0,006). Não houve diferença estatisticamente significante entre os Grupos II e III.

**Conclusão** A talidomida mostrou-se eficaz na redução da área da lesão e CPI dos implantes de endometriose experimental tanto na dose de 1 mg/kg/dia quanto na dose de 10 mg/kg/dia.

## Introduction

Endometriosis is characterized by the presence of endometrial tissue outside the uterine cavity, causing debilitating symptoms such as severe chronic pelvic pain and infertility in serious cases.^1^ The etiopathogeny of this disease is complex and multifactorial, involving genetic predisposition, environmental, anatomical and endocrine factors, and immunological changes.^2^

Once endometrial tissue is implanted, a constant stimulus of its development occurs in many cases, influenced by multiple factors such as escape from attack by the immunological system, changes in the local concentrations of hormones and inflammatory mediators, angiogenesis due to increased activity and levels of vascular endothelial growth factor A (VEGF-A) and proliferation of ectopic cells.^3^ ^4^ ^5^ ^6^

The estimate is that more than 70 million women all over the world are affected by endometriosis, which causes countless problems for the social, professional and marital life of these patients, with annual costs estimated at US$ 22 billion in the US alone. A reason for this high cost is the lack of efficient treatments of the disease.^3^

The treatment of endometriosis consists of conservative or radical surgery and drug therapy, with the former being associated with high rates of recurrence and significant morbidity.^7^ Different drug treatments are currently being proposed such as hormone therapy, anti-inflammatory agents and complementary therapies, although they tend to relieve symptoms rather than curing the disease. Thus, there still is the need to develop new medications for this purpose.^8^

Thalidomide has a potent antiangiogenic effect based on the negative regulation of vascular endothelial growth factor (VEGF). In addition, thalidomide functions as an immunomodulator and as an immunosuppressor and anti-inflammatory agent since it acts on the excessive synthesis of Tumor necrosis factor-alpha (TNF-α) and of other cytokines such as interleukin 6, which participate in the genesis of inflammatory pain.^9^ In view of this potential, under rigid regulation, thalidomide is currently being used for several diseases including erythema nodosum leprosum, Kaposi sarcoma associated with HIV-1, multiple myeloma, and advanced prostate cancer.^10^

Within this context, thalidomide acts in the endometrioses pathophysiological pathways. Thalidomide, although little explored, is a drug with great potential for the treatment of this disease, as it is able to act both in the control of progression and in the recurrence of the disease after the surgical treatment. Thus, the objective of the present study was to assess the action of thalidomide on the progression of implants and cell proliferation in endometriosis experimentally induced in rats using immunohistochemical PCNA labeling of eutopic and ectopic endometrium.

## Methods

The present study was conducted in the sector of experimental surgery of the University Hospital of Ribeirão Preto and in the Oncopathology laboratory of the Department of Pathology, Faculty of Medicine of Ribeirão Preto, University of São Paulo (FMRP-USP). The research project was approved by the Animal Experimentation Committee of FMRP-USP n° 060/2005.

Fifteen adult albino Wistar rats weighing ∼ 200 g were used. The animals were kept in appropriate cages under conditions of controlled temperature, humidity and lighting for 3 days before surgery, receiving water and food *ad libitum*. The animals were submitted to general anesthesia with 0.4 ml ketamine in combination with 0.2 ml xylestesin, followed by laparotomy for induction of endometriosis. The procedure was performed under strict antisepsis conditions, always by the same investigator. The pelvic cavity was opened by a median longitudinal incision of ∼ 2 cm at a distance of 2 cm from the pubis. A segment of ∼ 4 cm of the uterine horn was resected and the horn was closed. The resected uterine portion was immersed in 0.9% physiological saline at 4°C for 2 minutes and then incised longitudinally for the removal of two 5 × 5 mm fragments. The fragments of endometrial tissue were sutured to the peritoneum close to the reproductive tract of the animal using Vicryl 6.0 sutures, with the free endometrial surface facing the abdominal cavity; the surgical abdominal incision was then closed. No hormonal supplementation was administered before or after the surgery, with the procedure being performed during diestrus.^11^

Four weeks after surgery, the animals were divided into 3 groups: Group I (control – receiving an intraperitoneal Dimethyl Sulfoxide (DMSO) solution), Group II (10 mg/kg/day intraperitoneal thalidomide for 10 days), and Group III (1 mg/kg/day intraperitoneal thalidomide for 10 days). The rats were then euthanized, the widest diameters of the implants were measured, the total area of the lesion was calculated, the implants were excised, fixed in 10% formaldehyde and processed for paraffin embedding, and slides were mounted and stained with hematoxylin and eosin (H&E) to confirm the presence of endometrial tissue (identification of glandular epithelium and/or stroma).^11^

Histological sections (4 to 5 µm) were submitted to immunohistochemistry by the antigen-antibody reaction and the reaction was developed with a marker visible under the microscope. Deparaffinized and hydrated sections were recovered antigenically by incubation in buffered medium in a steam pot for 40 minutes. After cooling, the endogenous tissue peroxidases were removed by adding hydrogen peroxide, and horse serum was added to prevent nonspecific binding of the primary antibody. The slides were then incubated with primary antibodies obtained from Novocastra Laboratories Ltd. (Newcastle-upon-Tyne, UK). Cell proliferation was determined using PCNA (product code NCL-PCNA, PC10 clone) with nuclear labeling at a 1:200 dilution. The material was then incubated with the secondary antibody and submitted to the avidin-biotin step. The reaction was developed by treatment with Diaminobenzidine (DAB) (Sigma-Aldrich Inc., St. Louis, MI, USA) for 50 seconds, the material was counterstained with Harris H&E, and mounted on slides. All of the slides were evaluated by two pathologists experienced in immunohistochemistry who were not aware of the type of tissue to be analyzed.^11^

For evaluation of PCNA, which has nuclear labeling, we used a quantitative method, counting immunohistochemically labeled cells per 1,000 cells counted on the slide, with care taken to count cells in the four quadrants of each slide. Based on this calculation, we obtained the cell proliferation index (number of PCNA-labeled cells per 1,000 cells) for both the glandular and stromal tissues, which were analyzed separately.

Data were analyzed statistically with the GraphPad Prism 5.0 32-bit executable software (GraphPad Software Inc., San Diego, CA, USA) using the paired *t*-test, with the level of significance set at 5%.

## Results

Group I (control) had a mean lesion area of 69.2 mm^2^, whereas Groups II and III had significantly smaller mean areas of 49.4 mm^2^ (*p* = 0.023) and 48.6 mm^2^ (*p* = 0.006) compared with the control group, respectively, with no significant difference between them (*p* = 0.472). The control group also exhibited macroscopically more exuberant vascularization compared with the treated group.

**Fig. 1:**
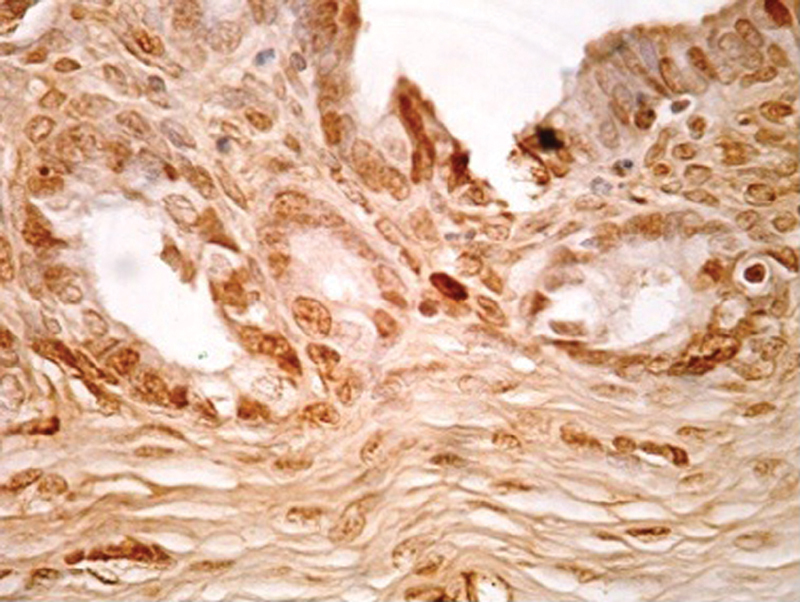
PCNA immunostaining of ectopic endometrium treated with a high thalidomide dose (Magnification: 40x).

**Fig. 2:**
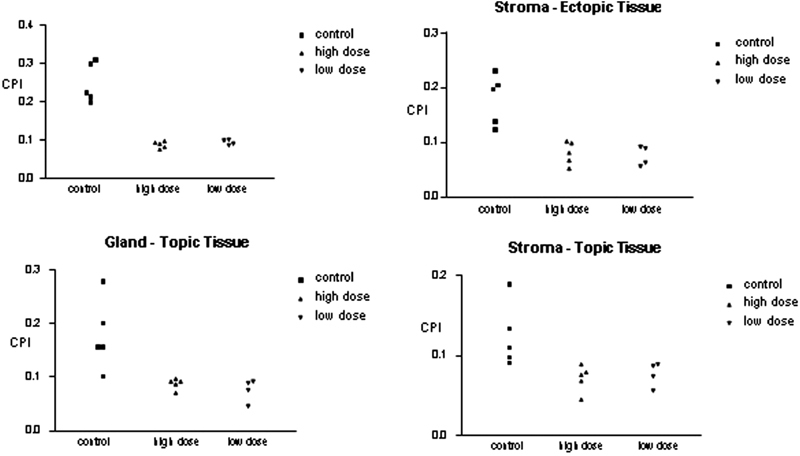
Cell proliferation index of topic and ectopic endometrial tissues.

Cell proliferation was determined using PCNA (Fig. 1). Group I (control) had a mean lesion CPI (gland and stroma) of 0.249 ± 0.051 and 0.179 ± 0.046, respectively. In contrast, Groups II and III had significantly lower lesion CPI values compared with the control group, of 0.088 ± 0.009 and 0.095 ± 0.007 (*p* < 0.001) for the gland and of 0.080 ± 0.021 and 0.075 ± 0.018 (*p* = 0.01) for the stroma, respectively, with no significant differences between them (*p* = 0.28) (Fig. 2).

A similar effect was observed on topic endometrial tissue, with a significant difference between the control and the two treatment groups. Group I (control) had a mean CPI of endometrium (gland and stroma) of 0.178 ± 0.066 and 0.124 ± 0.0396, respectively. In contrast, the CPI was significantly reduced in Groups II and III, with values of 0.088 ± 0.010 and 0.076 ± 0.021 (*p* = 0.008) for the gland and of 0.071 ± 0.039 and 0.077 ± 0.015 (*p* = 0.010) for the stroma, respectively. No significant difference in the CPI values of topic endometrial tissue were detected between Groups II and III (*p* = 0.12) (Fig. 2).

## Discussion

In most cases, the treatment of endometriosis involves the inhibition of ovarian function, with all its drawbacks.^12^ In view of the physiopathology of the disease and of the anti-inflammatory, immunomodulator and antiangiogenic action of thalidomide,^13^ this drug could be an option for the long-term treatment of endometriosis without interference with ovarian function.

In the present study, the implants of the treated groups (II and III) had significantly lower CPI and lesion areas than the control group. Comparison of these items between Groups II and III did not show significant differences, indicating that, even at low doses (Group III), thalidomide was effective in reducing cell proliferation and, consequently, the size of the implants. Similar results showing the antiproliferative effects of thalidomide was found in pancreatic cancer cell lines.^14^ Furthermore, thalidomide dithiocarbamate analogs exhibited significant antiproliferative action on human umbilical vein endothelial cells and MDA-MB-231 human breast cancer cell lines without causing cytotoxicity.^15^

It was also possible to perceive macroscopically that vascularization was reduced in the treatment groups, probably as a result of the antiangiogenic effect of thalidomide. This effect was first proven at the beginning of the decade of 1990,^16^ and today we know that thalidomide exerts its antiangiogenic effect in more than one manner, with emphasis on depletion of VEGF receptors^17^ and on the suppression of VEGF and basic fibroblast growth factor (bFGF) secretion.^18^ The growth and invasion of endometriosis lesions is absolutely dependent on neoangiogenesis. Within this context, VEGF-A is hyperexpressed in endometriosis and plays a fundamental role, inhibiting apoptosis and increasing the proliferation and migration of endothelial cells, representing an important target for the treatment of this disease.^19^

Two other studies conducted on an animal models have also obtained results similar to the present ones. Azimirad et al^20^ compared 2 groups of rats: a thalidomide group (*n* = 9; 22 mg/day) and a control group (*n* = 9; 0.5 mL 0.9% saline/day). These investigators also observed a significant reduction in implant volume and in the histopathology scores of the treatment group, in addition to a significant reduction in leukocyte, lymphocyte, VEGF-A and interleukin 6 (IL-6) counts in peritoneal fluid after treatment.

Bakacak et al^21^ compared a thalidomide group (*n* = 8; 100 mg/kg) and a control group (*n* = 8; 0.5 mL saline). In addition to observing a significant reduction of implant volume and of histopathology scores in the treatment group, they also detected a sigificant reduction of VEGF-A and myeloperoxidase in the peritoneal fluid.

A clinical study evaluated, with limitations, the administration of thalidomide to patients with endometriosis in a controlled pilot study. In that study, 10 women with grade IV endometriosis were treated with a Gonadotropin-releasing hormone (GnRH) analogue and thalidomide (300 mg/day) for at least 6 months, with GnRH being discontinued thereafter and only thalidomide being used. The study demonstrated the positive effects of thalidomide on the disease, with remission of pelvic pain and ovarian cysts being observed in 8 of the 10 patients. However, the cited study did not include a control group and did not assess the effects of thalidomide on the histopathology of endometriosis.^22^

The action of thalidomide was also assessed in cultured endometriotic stromal cells from eight women with moderate to severe endometriosis, with speculation that treatment with thalidomide reduced the expression of interleukin 8 (IL-8) by reducing TNF-α-induced nuclear factor kappa B (NF-κB) activation, with this action being of fundamental importance for the reduction of inflammation.^23^ Since inflammation is crucial for the pathogenesis of endometriosis, with emphasis on the TNF-α cytokine, whose expression is increased in the tissues of patients with endometriosis, and which also directly influences the expression of estrogen receptors,^24^ the interference of thalidomide with this pathway may explain part of the findings of the present study.

The present results, taken together with those obtained in the few published studies, demonstrate the potential of thalidomide for the treatment of endometriosis, supporting the hypothesis that this drug causes a regression of the evolution of endometriosis and underscoring the need for further research conducted with due caution in view of the teratogenic history of this drug.^25^

## Conclusion

Thalidomide proved to be efficient in reducing the lesion area and the CPI of peritoneal endometriotic implants in rats, both at the dose of 1 mg/kg/day and at the dose of 10 mg/kg/day, with the lower dose being as effective as the dose of 10 mg/kg/day, which is known to be teratogenic. Thus, thalidomide should be considered as a potential drug for the treatment of endometriosis in women.
